# Bioactive lipids in intervertebral disc degeneration and its therapeutic implications

**DOI:** 10.1042/BSR20192117

**Published:** 2019-10-11

**Authors:** Undurti N. Das

**Affiliations:** UND Life Sciences, 2221 NW 5th St, Battle Ground, WA 98604, USA and BioScience Research Center and Department of Medicine, GVP Medical College and Hospital, Visakhapatnam 530048, India

**Keywords:** degeneration, eicosanoids, inflammation, intervertebral disc, lipoxin A4, prostaglandin E2

## Abstract

Intervertebral disc (IVD) degeneration is not uncommon. It is estimated that approximately >60% of individuals above the age of 40 years suffer from IVD degeneration. Shan et al. showed that hyperglycemia can enhance apoptosis of anulus fibrosis cells in a JNK pathway and p38 mitogen-activated protein kinase (MAPK) pathway dependent fashion. Recent studies showed that IVD degeneration could be an inflammatory condition characterized by increased production of matrix metalloproteinases, TNF-α, nitric oxide, IL-6, IL-17, IL-9, and prostaglandin E2, and decreased formation of anti-inflammatory molecules such as lipoxin A4. This imbalance between pro- and anti-inflammatory molecules seem to activate JNK pathway and p38 MAPK pathway to induce apoptosis of anulus fibrosis and nucleus pulposus cells. The activation of production of PGE2 (due to activation of COX-2 pathway) seems to be dependent on p38/c-Fos and JNK/c-Jun activation in an AP-1-dependent manner. These results imply that suppressing pro-inflammatory events in the disc by either augmenting anti-inflammatory events or suppressing production of pro-inflammatory molecules or both may form a logical step in the prevention and management of IVD degeneration.

## Introduction

Intervertebral disc (IVD) degeneration is an age-related condition that occurs as a result of one or more of the discs between the vertebrae of the spinal column deteriorates or breaks down, leading to pain. More often than not there could occur weakness, numbness and pain that radiates down the leg. It needs to be mentioned here that despite its name, degenerative disc disease is not a disease, but a natural occurrence that happens with aging though this debated. The rubbery discs between the vertebrae normally allow for flexing and bending of the back, like shock absorbers. They may become worn with time and age and thus, they may no longer offer as much protection as before.

**Figure 1:**
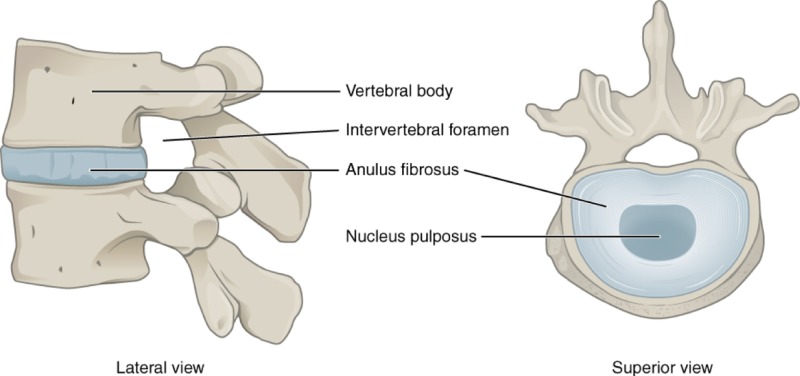
Lateral and superior view of IVD in position between two vertebrae

**Figure 2:**
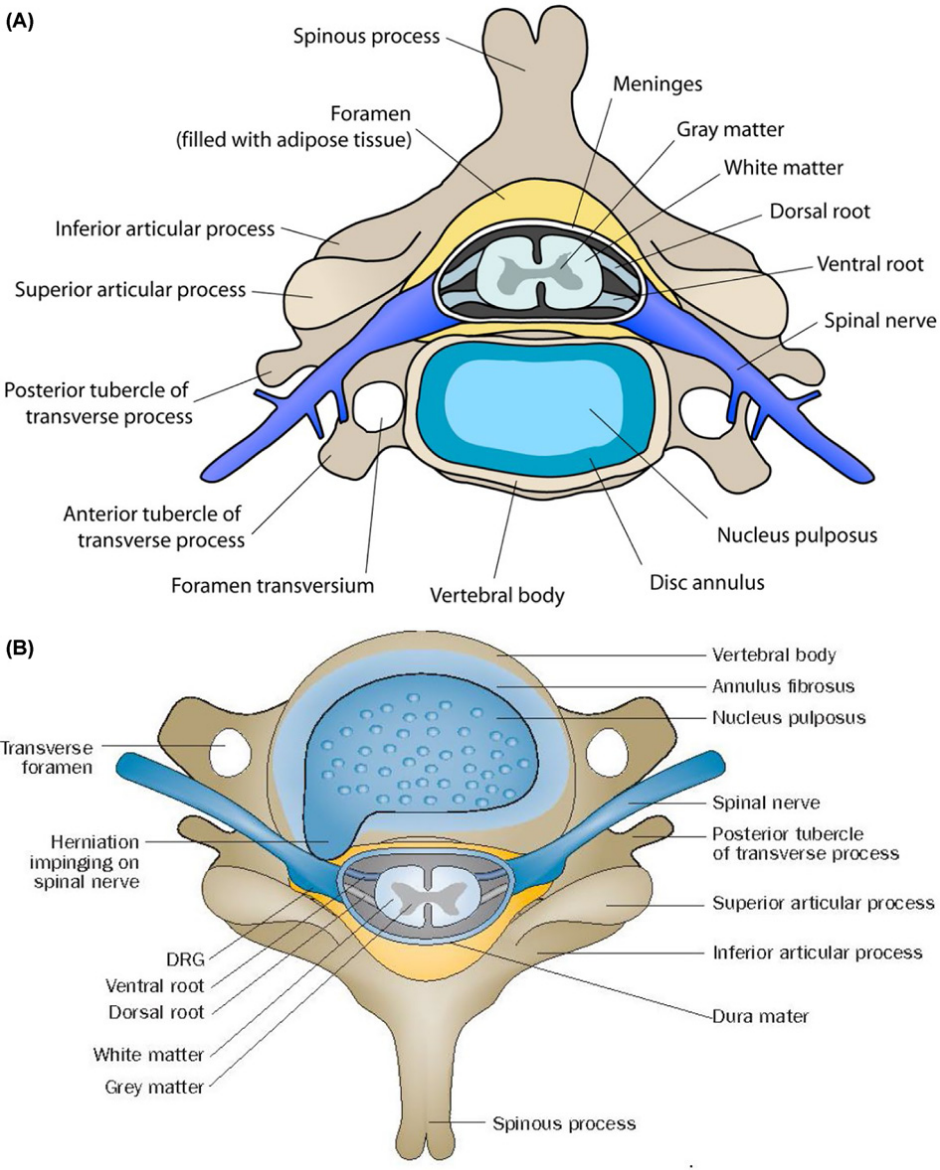
Cross section of vertebra with intervertebral disc (**A**) Scheme showing relationship among various vertebral structures, a herniated disc and spinal nerves. Extrusion of the nucleus pulposus through anular fissures could impinge on a spinal nerve(s) and induce inflammatory response that ultimately results in radicular pain. DRG = Dorsal root ganglion; IVD = Intervertebral disc. Taken from: Makarand V Risbud, Irving Milton Shapiro. Nature Reviews Rheumatology 2014; DOI: 10.1038/nrrheum.2013.160.

IVDs, also known as intervertebral fibrocartilage or spinal discs, provide the much-needed padding between the vertebrae of the spine. They have an elastic structure, made of fibrocartilage tissue (Figures 1 & 2).

The outer part of the disc is known as the anulus fibrosus. It is tough and fibrous, and it consists of several overlapping layers. The inner core of the disc is the nucleus pulposus. It is soft and gelatinous.

The IVD not only functions to separate the vertebrae from each other but also provides the much-needed surface for the shock-absorbing gel of the nucleus pulposus that functions to distribute hydraulic pressure in all directions within each IVD under compressive loads. The nucleus pulposus consists of large vacuolated notochord cells, small chondrocyte-like cells, collagen fibrils, and aggrecan, a proteoglycan that aggregates by binding to hyaluronan. Attached to each aggrecan molecule are glycosaminoglycan (GAG) chains of chondroitin sulfate and keratan sulfate. Increasing the amount of negatively charged aggrecan increases oncotic pressure, resulting in a shift of extracellular fluid from the outside to the inside of the nucleus pulposus. The amount of GAGs (and hence water) decreases with age and degeneration [1].

The anulus fibrosis surrounds the nucleus pulposus of the IVD. The nucleus pulposus and the inner anulus fibrosus are separated from the bony vertebral endplate by a thin layer of hyaline cartilage that consists of proteoglycans and collagen fibers. It is noteworthy that the chemical composition of the fibrocartilaginous endplates’ is similar to the other components of the IVD implying that there is unlikely to be any barrier for diffusion of nutrients through the endplates, which is a key pathway for nutrient support to the IVDs. This is important since nutrients reach IVDs via diffusion, and only to a minor extent by bulk transport since disc has no or only a very limited number of blood vessels that are restricted to the outer anulus.

The central nucleus pulposus mainly consists of proteoglycans (about 50% of the dry weight of the nucleus), which represent long chains of GAGs linked to proteins. Proteoglycans are highly negatively charged allowing them to attract positively charged cations from the interstitial fluid and thus, they have the capacity to attract and imbibe water, which amounts to approximately 80% of the total weight of the nucleus pulposus in young individuals. The rest of the 20% of the dry weight of the nucleus pulposus is collagen type II that holds the proteoglycans together. The nucleus pulposus also contains small amounts of collagen type I, elastin fibers, and other non-collagenous proteins.

The circular anulus fibrosis that surrounds the nucleus pulposus consists of up to 20 layers of circular collagen type I-rich sheets, called lamellae that are interconnected by radial collagen bundles and the rest of the space is filled with proteoglycans. Compared with the nucleus pulposus, the anulus fibrosus of a healthy disc contains significantly fewer proteoglycans and consequently approximately 10% less water, but more than double the amount of collagens. In contrast with the nucleus pulposus, where collagen type II is predominant, the main collagen of the anulus fibrosus is type I, which is more suitable to transmit and sustain tensile forces [2].

**Figure 3:**
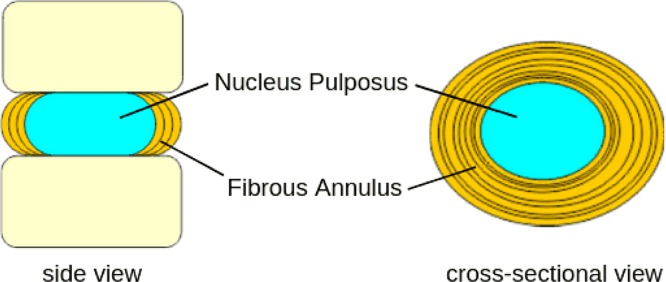
Distribution of load in the IVD

**Figure 4:**
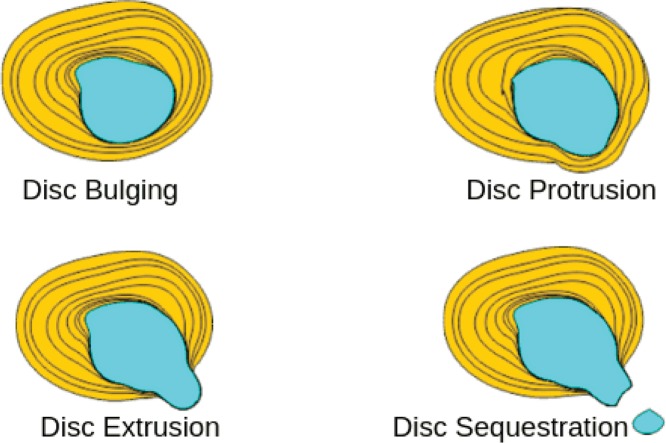
Four stages of disc herniation

The jelly-like soft nucleus pulposus and tough exterior anulus fibrosus lies between two vertebrae is called as the IVD that renders spine flexible and acts as a shock absorber (Figure 3). It is evident from the preceding discussion that integrity of anulus fibrosis is important to prevent nucleus pulposus from bulging, protrusion, extrusion, and sequestration as shown in Figures 2A and 4. Changes that lead to IVD degeneration include: loss of fluid that normally consist of up to 90% of its total fluid content. This loss of fluid causes the disc thinner, the distance between vertebrae becomes smaller that causes the disc to be less effective as a shock-absorber. Over a period of time, the disc structure itself could change due to small tears or cracks in the outer layer of the disc due to repeated use. This results in seeping of the gelatinous material from the nucleus pulposus and hence, the disc may break into fragments leading to less padding between the vertebrae making the spine less stable (Figure 4). These degenerative changes can cause compensatory development of osteophytes or bone spurs that can press against the spinal roots causing pain. The bulging disc called as a herniated disc can press on the nerves and cause pain, weakness, and numbness.

It is estimated that before age 40, approximately 25% of people show evidence of disc degeneration. Beyond age 40, more than 60% of people show evidence of disc degeneration on MRI and paradoxically, these degenerative changes do not correlate to pain and are considered as a part of normal ageing process [3–5].

The integrity of anulus fibrosis and its ability to prevent the seeping of the gelatinous material from the nucleus pulposus depends on the health of the anulus fibrosis (AF) cells. Thus, the balance between the rate of apoptosis of AF cells and their ability to divide and replace the apoptotic cells is crucial to the integrity of anulus fibrosis and the prevention of disc degeneration. In this context, the study by Shan et al. [6] is noteworthy which showed that hyperglycemia increased anulus fibrosis cell apoptosis in a JNK and p38 mitogen-activated protein kinase (MAPK) pathway dependent manner implying that efforts directed to inhibit this pathway may form a new approach to prevent disc degeneration.

## IVD disease as a pro-inflammatory condition

Despite the general belief that IVD disease is a degenerative disorder, there is now substantial evidence to suggest that it could be a pro-inflammatory condition. It is likely that IVD cells exhibit diverse biologic responses to mechanical stimuli, depending on the loading type, magnitude, duration and anatomic zone of cell origin. It is proposed that the inner most cells respond to low-to-moderate magnitudes of static compression, osmotic pressure or hydrostatic pressure with an increase in anabolic cell responses, whereas higher magnitudes of loading may produce catabolic responses in the form of elevated protease gene or protein expression or activity. It is predicted that the responses of the IVD cells respond in a differentia manner to various micromechanical stimuli depending on their location, amount of exposure to the mechanical stimuli, duration of exposure, frequency of exposure, etc. It is likely that large hydrostatic pressures, but little volume change, are predicted to occur for cells of the nucleus pulposus during compression, while the cells of the anulus fibrosus may experience deformations in tension or compression during matrix deformations. In general, it is possible that the cells of the nucleus pulposus and inner portion of the anulus fibrosus show comparable micromechanical stimuli *in situ* and may respond more similarly than cells of the outer portion of the anulus fibrosus. But little is known about the mechanisms that regulate cellular responses to intervertebral mechanobiology. It is predicted that mechanical factors may regulate responses of the IVD cells through mechanisms involving intracellular Ca^2+^ transients and cytoskeletal remodeling that are likely to be involved in the regulation of downstream events such as gene expression and post-translational biosynthesis that could include genetic factors, cytokines, and inflammatory mediators that ultimately determine the regulation of IVD degeneration/disease [7]. Some of these assumptions are supported by the results of a study wherein the rabbit IVD explants *in vitro* were subjected to unconfined uniaxial compression, static compression load suppressed gene expression for collages and aggrecan in the disc, whereas dynamic compression enhanced anabolic change as evidenced by increase in the expression of type I and type II collagen and aggrecan with distinct regional differences in the responses to mechanical loading between the anulus fibrosus and nucleus pulposus. All loading conditions produced marked histologic changes, and a significant increase in the expression of matrix metalloproteinases (MMP1, MMP2, MMP3, and MMP13), IL-1β and TNF-α and a substantial increase in TUNEL positive cells in the intervertebral cells [8,9].

Furthermore, cells derived from nucleus pulposus and anulus fibrosus of Sprague–Dawley rat tails cultured with or without cyclic mechanical stress (CMS) in the presence or absence of inflammatory stimulus increased prostaglandin E2 (PGE2) and CMS, and inflammatory stimulus synergistically enhanced PGE2 synthesis of both cell types. It is noteworthy that anulus fibrosus cells showed a stronger reactivity to these stimuli than nucleus pulposus cells. The expression of cyclo-oxygenase-2 (COX-2, which is needed for PGE2 synthesis) mRNA of anulus fibrosus cells correlated to the amount of PGE2, whereas COX-2 mRNA was constitutively expressed in nucleus pulposus cells. These results imply that the role of COX-2 might be different between nucleus pulposus and anulus fibrosus, whereas phospholipase-A2 IIA (that is needed for the release of arachidonic acid (AA), the precursor of PGE2 from the cell membrane lipid pool) mRNA was constitutively expressed in both cell types [10]. These *in vitro* and animal studies [9,10] were reinforced by the studies performed with cultured normal and herniated human IVD specimens, it was reported that normal, control disc specimens significantly increased their production of matrix metalloproteinases, nitric oxide, IL-6, and PGE2 in response to IL-1β, whereas herniated lumbar and cervical discs spontaneously released increased levels of these biochemical molecules and showed further increase in the production of nitric oxide, IL-6, and PGE2 in response to IL-1β. Surprisingly, blocking of nitric oxide synthesis in IL-1β stimulated disc cells produced a large increase in the production of IL-6 suggesting a feedback regulation amongst IL-1, IL-6, and NO with NO having an inhibitory action on IL-6 production [11–13]. It was reported that suppression of NO production increased proteoglycan synthesis in the IVD specimens in a dose-dependent fashion, whereas increased NO generation suppressed proteoglycan synthesis. It is interesting that three-atmosphere hydrostatic pressure stimulated the proteoglycan synthesis, whereas 30-atm pressure inhibited proteoglycan synthesis rates. In contrast, at 3 atm, NO production was decreased whereas at a pressure of 30 atm, NO production was increased [14]. These results attest to the fact that (1) human herniated lumbar disc cultures spontaneously produce NO; (2) NO inhibits proteoglycan synthesis in the IVD; and (3) NO inhibits IL-6, a pro-inflammatory cytokine, production. Thus, NO seems to play an important role in the regulation of disc cell metabolism under mechanical stress and in the pathophysiology of IVD disease. In addition, large numbers of peripheral blood mononuclear cells were found to be attached to the surfaces of extruded discs, and these contained higher amounts of MMP-1 and MMP-3 compared with the controls. Furthermore, significant enhancement of MMP-1 and MMP-3 mRNA expression was noted in the disc-derived cells stimulated with cytokines IL-6, IL-1β, and TNF-α [15]. The increased macrophage infiltration of the discs seems to be associated with increased macrophage produced MMPs that are needed for proteoglycan degradation, loss of wet weight, and subsequent disc degeneration. The MMPs seem to be needed for the release of TNF-α from macrophages, whereas TNF-α is essential for the induction of MMPs in disc cocultures, which, in turn, is essential for macrophage infiltration. Thus, there is an extensive cross-talk between macrophages and chondrocytes in herniated disc resorption that is made possible by MMPs, cytokines and NO and other soluble bioactive factors [10–18]. This cross-talk is further influenced by the observation that TNF-α-induces vascular endothelial growth factor (VEGF) production in anulus fibrosis cells in culture primarily through the NF-κB pathway. The angiogenic activity stimulated by VEGF could be inhibited by NF-κB inhibitors or anti-VEGF antibody. Since neovascularization is essential for herniated disc resorption, these results imply that inhibition or blocking of TNF-αay aid in the prevention of IVD degeneration and resorption [19].

## IL-1 and TNF-α activate JNK or p38 MAPK pathway

c-Jun N-terminal kinases (JNKs) that belong to mitogen-activated protein kinase family, bind and phosphorylate c-Jun on Ser-63 and Ser-73 within its transcriptional activation domain. JNKs respond to stress stimuli including cytokines, ultraviolet irradiation, heat shock, and osmotic shock (including mechanical pressure as seen in subjects with IVD disease). JNKs play a role in T cell differentiation and cellular apoptosis pathway and contribute to inflammatory responses.

P38 mitogen-activated protein kinases are a class of MAPKs that respond stress stimuli: cytokines, ultraviolet irradiation, heat shock, and osmotic shock, and are involved in cell differentiation, apoptosis, and autophagy. Thus, there is a very close relationship and overlapping actions between JNKs and p38 MAPK pathway and they interact with each other [20,21].

Nucleus pulposus cells when exposed to IL-1 or TNF-α in the presence of p38 MAPK inhibition showed decreased PGE2, NO and IL-6 production and increased the ratio of tissue inhibitor of matrix metalloproteinase-1 (TIMP-1) to MMP-3. These results indicate that inhibition of p38 MAPK can be used as a strategy to suppress inflammation and associated disc matrix catabolism as a viable therapeutic approach to slow IVD degeneration [22,23]. Hyperbaric oxygen decreased expression of IL-1β, increased the gene expression of aggrecan and type II collagen, suppressed the phosphorylation of p38 MAPK, decreased NO, PGE-2, and MMP-3, and increased TIMP-1 expression in nuclear pulposus cells lending support to the concept that IL-1β and the p38 MAPK signal play a significant role in the inflammatory and catabolic changes associated with disc degeneration and hyperbaric oxygen treatment may be of therapeutic benefit in this condition [24–26].

In addition to IL-1, IL-6, and TNF-α other pro-inflammatory cytokines that seem to be involved in disc degeneration process are IL-17 and IL-9. Nucleus pulposus cells isolated from patients undergoing IVD degeneration showed increased expression of COX-2 and PGE2 production due to activation of MAPK/activating protein-1 (AP-1) pathway, p38 kinase, and JNK in response to IL-17A, indicating a role for cytokines IL-17 in this disease [27]. Similarly, nucleus pulposus cells from subjects with IVD disease showed enhanced expression of IL-9 and TNF-α. IL-9 significantly up-regulated TNF-α and PGE2 secretion by these cells suggesting a critical role for this cytokine in IVD degeneration [28].

Based on these results [10–28], it is reasonable to propose that IVD degeneration can be considered as an inflammatory disease in which IL-1, IL-6, TNF-α, NO, IL-17, IL-9, PGE2, metalloproteinases, activation of JNK, and p38 MAPK pathways occur leading to an imbalance in Bax/BCL-2 and activation of caspases that ultimately results in apoptosis of nucleus pulposus and anulus fibrosis cells. These events ultimately lead to degeneration of IVD. These adverse changes seem to be exaggerated by hyperglycemia as shown by Shan et al [6].

## Lipoxin A4, epoxyeicosatrienoic acid, and soluble epoxide hydrolase in IVD degeneration

In this context, it is noteworthy that both IVD degeneration and hyperglycemia (diabetes mellitus) may have some common abnormalities with regard to essential fatty acid metabolism that may be relevant to the role of these lipids in the pathobiology of IVD degeneration and developing newer therapeutic strategies.

**Figure 5:**
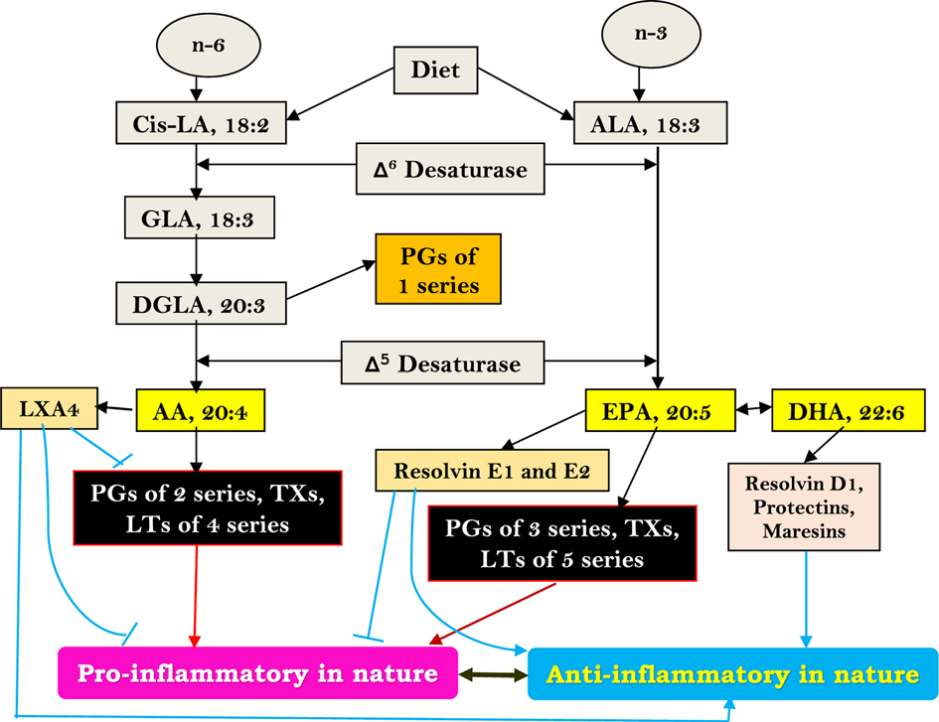
Gamma-linolenic acid, DGLA, and AA from linoleic acid and eicosapentaenoic acid and DHA Dietary essential fatty acids’ (LA and ALA) metabolism. LA = Linoleic acid; GLA = Gamma-linolenic acid; DGLA = Dihomo-gamma-linolenic acid; AA = Arachidonic acid; ALA = Alpha-linolenic acid; EPA = Eicosapentaenoic acid; DHA = Docosahexaenoic acid; PGs = Prostaglandins; LTs = Leukotrienes; TXs = Thromboxanes; LXA4 = Lipoxin A4. It may be noted here that PGs and TXs of three series and LTs of five series formed from EPA are less pro-inflammatory compared with two series PGs and TXs and four series LTs formed from AA. LXA4, resolvins, protectins, and maresins can inhibit formation of pro-inflammatory PGs, TXs and LTs.

**Figure 6:**
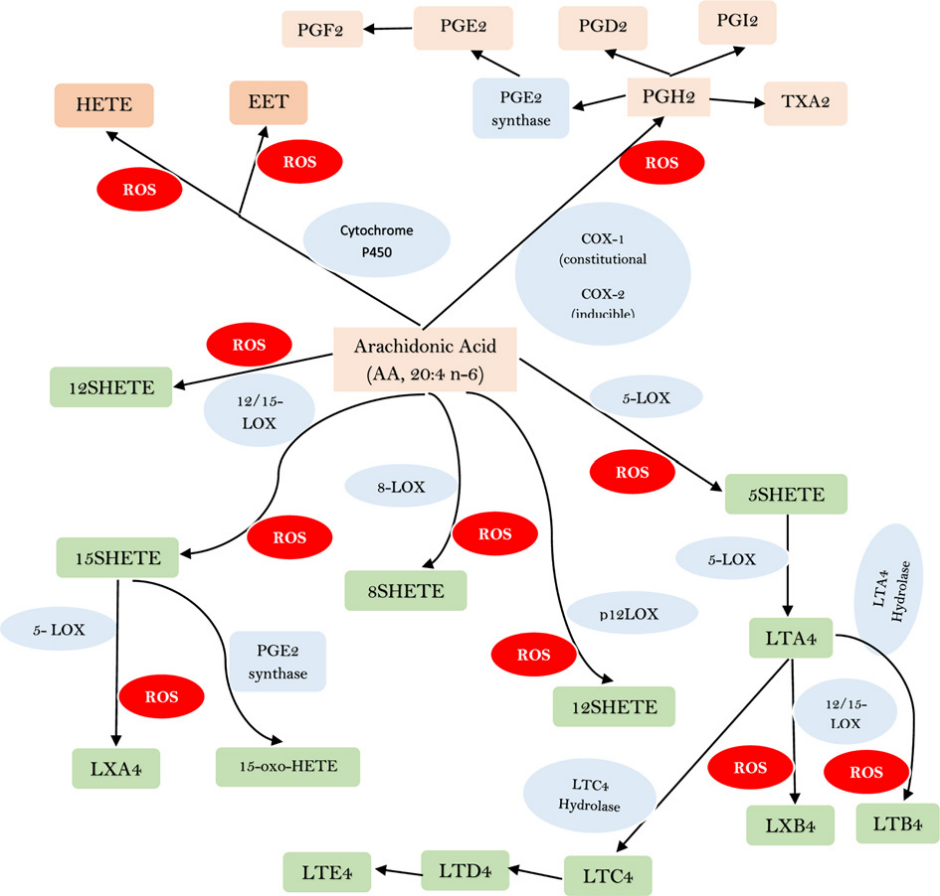
AA metabolism through COX, LOX and p450 pathways Both EPA and DHA may follow similar metabolic pathways. COX = Cyclo-oxygenase; LOX = Lipoxygenase; ROS = Reactive oxygen species; HETE = Hydroxyeicosatetraenoic acid; EET = Epoxyeicosatrienoic acid.

Dietary linoleic acid (LA) and α-linolenic acid (ALA) are converted to their respective long-chain metabolites namely gamma-linolenic acid (GLA), dihomo-GLA (DGLA), and AA from LA, and eicosapentaenoic acid (EPA) and docosahexaenoic acid (DHA) from ALA possibly due to the action of same set of enzymes (Figure 5). DGLA forms the precursor to prostaglandin E1 (PGE1), whereas AA and EPA form precursors to respective prostaglandins leukotrienes and thromboxanes as shown in Figures 5 and 6. In general, PGs, LTs, and TXs have pro-inflammatory actions. Lipoxin A4 formed from AA and resolvins of E series from EPA and resolvins of D series, protectins and maresins formed from DHA have potent anti-inflammatory actions [29]. The precursors for various eicosanoids (all PGs, LTs, TXs, LXA4, resolvins, protectins and maresins, and other similar metabolites), termed as polyunsaturated fatty acids (PUFAs), are derived from the cell membrane lipid pool and form an important constituent of the cell membranes. Both PUFAs and their metabolites have many actions that are critical to several physiological and pathological processes. It is likely that under physiological conditions a balance is maintained between pro- and anti-inflammatory eicosanoids and when this balance is tilted more toward pro-inflammatory metabolites, inflammatory events could be initiated and progressed.

**Figure 7:**
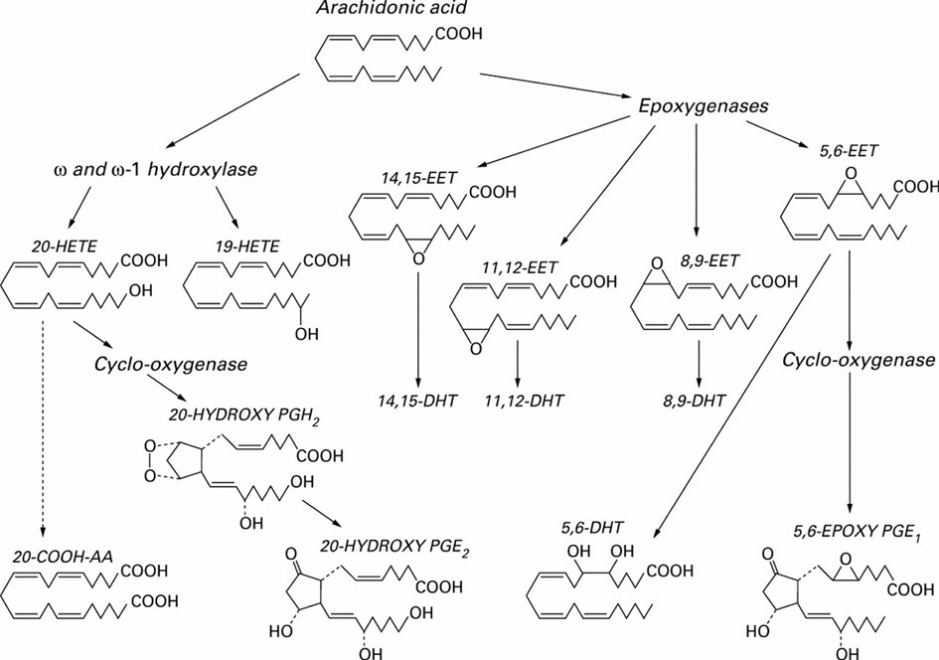
AA metabolism by cytochrome P450 dependent mono-oxygenases to ω- and ω-1-hydroxyeicosatetraenoic acids (HETEs), epoxyeicosatrienoic acids (epoxides, EETs), and dihydroxyeicosatrienoic acids (diols, DHTs) 20-HETE and 5,6-EET can be converted by cyclo-oxygenase to analogues of prostaglandins. Note that there are at least 4 types of EETS: 5, 6-EET; 8, 9-EET; 11, 12-EET and 14, 15-EET are formed from AA that may have overlapping actions. Both EPA and DHA may undergo similar metabolic pathways.

The observation that increased amounts of PGE2, a pro-inflammatory eicosanoid derived from AA, are generated by cells of nucleus pulposus and anulus fibrosis [11–13] suggests that there could occur simultaneously decreased synthesis of anti-inflammatory metabolites from PUFAs. This is supported by the observation that LXA4 is of significant benefit in a rat model of non-compressive lumbar disc herniation by inhibiting ERK, JNK and NF-kB/p65, and pro-inflammatory cytokines IL-1β and TNF-α, and up-regulating the expression of anti-inflammatory cytokines: TGF-β and IL-10 [30]; and 14, 15-EET (epoxyeicosatetraenoic acid, also derived from AA (Figure 7 for the metabolism of EETs) protected rat nucleus pulposus cells against death induced by TNF-α *in vitro* by inhibiting the NF-κB pathway and local administration of 14,15-EET prevented IVD degeneration [31]. It is noteworthy that soluble epoxide hydrolase set of enzymes can metabolize EETs and thus, limit their beneficial actions especially in the prevention of IVD degeneration. Hence, inhibiting epoxide hydrolase enzymes to enhance the half-life of EETs may form a new approach to prevent and manage IVD degeneration. It is evident from these studies [11–13,30,31] that a delicate balance exists between pro-inflammatory PGE2 (and possibly, other pro-inflammatory eicosanoids) and anti-inflammatory LXA4 (and possibly, resolvins, protectins, and maresins) and EETs and if the balance could be tilted more toward LXA4 and EETs may prevent IVD degeneration. It is interesting to note that both PGE2 and LXA4 (and resolvins, protectins, and maresins) suppress IL-1β, IL-6, TNF-α and metalloproteinases expression and thus, bring about their anti-inflammatory actions. In contrast, IL-1β, IL-6, and TNF-α augment COX-2 expression and enhance the production of PGE2 and other pro-inflammatory eicosanoids. The paradoxical action of PGE2 to suppress IL-1β, IL-6, and TNF-α production yet possess pro-inflammatory action suggests that there is a cross-talk between PGE2 and cytokines, and is meant to limit inflammatory process. It is paradoxical that AA forms the precursor of both PGE2 and LXA4, implying that regulation of AA metabolic pathway is crucial in the regulation of inflammatory process and consequent diseases. It was reported that in many inflammatory conditions in which increased PGE2 production is seen are also characterized by AA deficiency and reduced production of LXA4 (reviewed in [29,32–37] ). In these AA deficiency states, administration of AA enhances LXA4 production with no change in PGE2 synthesis [29,38,39].

This is further supported by our studies that showed that hyperglycemia due to type 1 and type 2 diabetes mellitus induced by chemicals, such as alloxan and streptozotocin, in experimental animals could be prevented by various n-3 and n-6 fatty acids, especially AA and LXA4 [40–43]. Furthermore, it was noted that experimental animals with type 1 and type 2 diabetes mellitus have low plasma and tissue levels of AA and LXA4 [40–45], and increased plasma levels of PGE2 [46–48] implying that diabetes is an inflammatory condition. Thus, it appears that both IVD degeneration and diabetes mellitus are inflammatory conditions and both are benefited by anti-inflammatory strategies. This may explain as to why hyperglycemia (diabetes mellitus) exaggerates IVD degeneration as shown by Shan et al [6].

## Conclusion and future perspective

**Figure 8:**
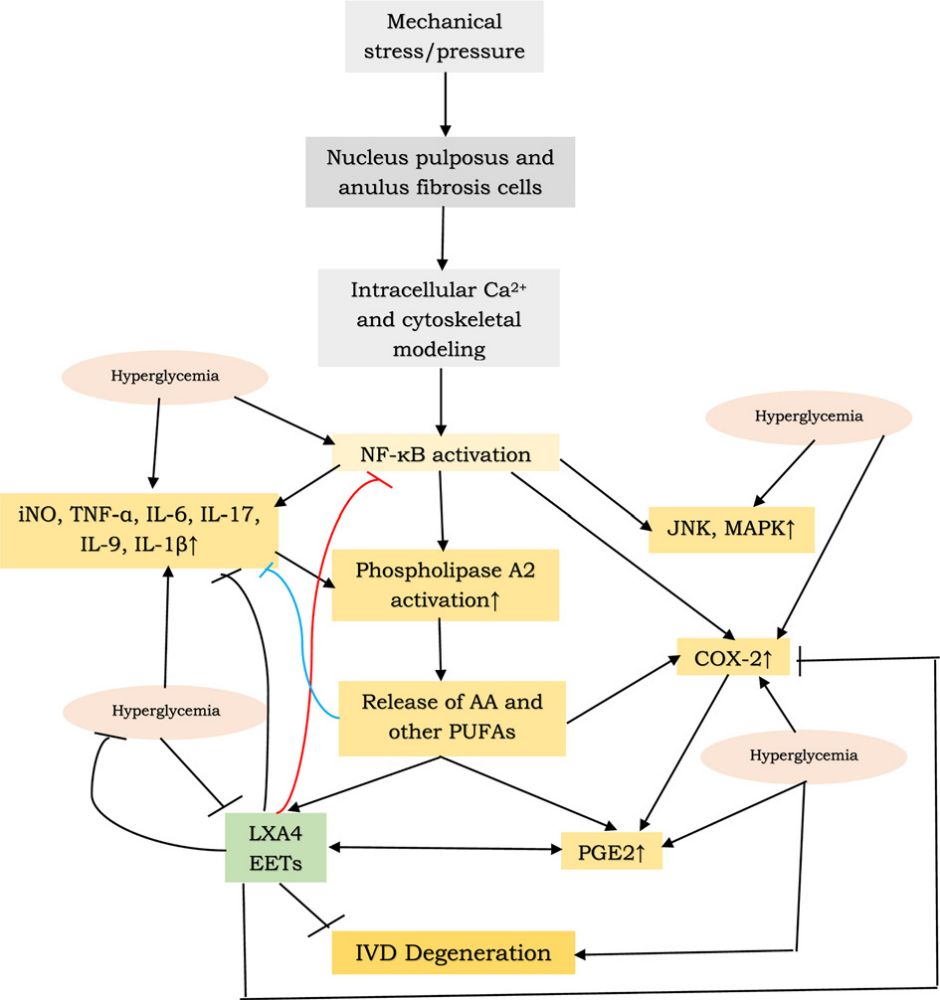
Scheme showing sequence of events that could lead to IVD degeneration and the role of various molecules in this process Hyperglycemia has pro-inflammatory actions and thus, exaggerates IVD degeneration. iNO = inducible nitric oxide.

Based on the preceding discussion, it is suggested that IVD degeneration is an inflammatory condition ( Figure 8). In view of the pro-inflammatory nature of IVD prolapse, studies have been conducted employing anticytokine therapies (such as etanercept, adalimumab, and infliximab that are anti-TNF monoclonal antibodies and tocilizumab, an anti-IL-6 antibody), but the results have given conflicting results. In this context, use of LXA4 and other anti-inflammatory bioactive lipids, such as resolvins, protectins, and maresins, may be worth trying since they can inhibit the production and action of IL-6, TNF-α and other pro-inflammatory cytokines. Since IVD degeneration is more common in elderly subjects who are likely to have diabetes mellitus that may exaggerate the disease and may worsen the prognosis. In this context, it will be highly relevant to study the expressions of enzymes desaturases, COX, LOX, soluble epoxide hydrolases, and the concentrations of PGE2, LXA4 and other pro- and anti-inflammatory eicosanoids and their precursors in the pathobiology of IVD degeneration and how they could influence the survival, proliferation and resilience of cells of nucleus pulposus and anulus fibrosis. Studies pertaining to the cross-talk amongst pro- and anti-inflammatory cytokines, eicosanoids and their precursors is needed. It is possible that injections of LXA4 and other anti-inflammatory resolvins, protectins and maresins at the site of IVD degeneration could be of significant benefit in the arrest of IVD degeneration. The possibility that oral supplementation of AA and other PUFAs may be of significant benefit in the prevention and management of IVD degeneration needs to be explored (since we observed that oral AA is as potent as that of intraperitoneal AA in preventing DM [42,43]. It is important to study whether local administration of EET and soluble epoxide hydrolase inhibitors are of benefit in arresting IVD degeneration. The current evidences highlight how understanding the molecular pathological events could lead to the development of newer preventive and therapeutic strategies that ultimately benefit the patient(s).

## Abbreviations

AA: arachidonic acid
ALA: α-linolenic acid
CMS: cyclic mechanical stress
COX-2: cyclo-oxygenase-2
DGLA: dihomo-gamma-linolenic acid
DHA: docosahexaenoic acid
EET: epoxyeicosatrienoic acid
EPA: eicosapentaenoic acid
GAG: glycosaminoglycan
GLA: gamma-linolenic acid
IVD: Intervertebral disc
LA: linoleic acid
MAPK: mitogen-activated protein kinase
PGE2: prostaglandin E2
VEGF: vascular endothelial growth factor
